# Agronomic Performance and Nutritive Value Evaluation of Desho Grass Varieties Under Supplementary Irrigation in Western Oromia, Ethiopia

**DOI:** 10.1155/tswj/8834746

**Published:** 2026-07-03

**Authors:** Fikre Dereba, Zemene Worku, Diriba Geleti

**Affiliations:** ^1^ Department of Animal Feed and Nutrition Research, Oromia Agricultural Research Institute, Adami Tulu Agricultural Research Center, Batu, Oromia, Ethiopia, iqqo.org; ^2^ Department of Animal Sciences, College of Agricultural and Veterinary Medicine, Jimma University, Jimma, Ethiopia, ju.edu.et; ^3^ Department of Animal Feed and Nutrition Research, Ethiopia Institute of Agricultural Research, Addis Ababa, Ethiopia

**Keywords:** desho grass, dry matter yield, in vitro digestibility, nutritive value, varieties

## Abstract

The study was conducted to evaluate the agronomic performance, forage yield, and nutritive values of desho grass (*Pennisetum glaucifolium*) varieties under supplementary irrigation at Dambi Dollo University experimental site, Western Ethiopia. The varieties (Areka/DZF #590, Kindu kosha‐1/DZF #591, and Kulumsa/DZF #592) were arranged in a randomized complete block design with four replications. The crop water requirements 8.0 model, local climate data, forage data, and soil data were used to determine desho grass water requirements and irrigation schedules. The parameters such as agronomic performance, yield, chemical composition, and in vitro digestibility of the forage samples at 105 days after planting were determined following the standard procedures. The results showed significant differences (*p* < 0.05) among varieties in most agronomic parameters except plant height and leaf width. The highest dry matter and crude protein yields were recorded from Areka/DZF #590 (12.64 and 1.35 t/ha) followed by Kulumsa/DZF #592 (11.63 and 1.12 t/ha). The chemical composition of varieties differed significantly except for hemicellulose. The in vitro digestibility of Areka/DZF #590 (624.7 g/kg) was significantly higher than Kulumsa/DZF #592 (584.3 g/kg) and Kindu kosha‐1/DZF #591 (580.9 g/kg). In conclusion, the Areka (DZF #590) desho grass variety showed superior dry matter yield and good nutritive value under supplementary irrigation conditions. Therefore, this variety is suitable and recommended for use as animal feed in the study area.

## 1. Introduction

The Ethiopian livestock population is estimated to be 70 million cattle (Bos indicus), 42.9 million sheep, 52.5 million goats, 2.15 million horses, 10.80 million donkeys, 0.38 million mules, and about 8.1 million camels, as well as 57 million poultry [1]. Despite Ethiopian abundant livestock resources and many roles, livestock productivity has remained low and unable to meet the growing demands of the country′s population [2]. Various factors contribute to low productivity in Ethiopia, such as low feed quality, fluctuating seasonal availability of feed, poor genetics, disease, and limited accessibility to inputs and services [3].

Inadequate nutrition and feeding are the major challenges facing livestock production [4]. This is due to climate changes, shrinkage of grazing areas, land tenure, border conflict, weed, and bush encroachment, soil degradation, and unavailability of seed for improved forage varieties as reported by Mengistu et al. [5] as well as other factors such as inadequate management practices, feeding mostly crop residue, and insufficient animal feed preservation practices [4]. The prolonged dry season and the absence of irrigation systems also impose additional challenges to the development and appropriate management of perennial forage crops [6].

Ethiopia has significant potential for forage production through small‐scale irrigation, with approximately 31% of the country being highly suitable for growing desho grass (*Pennisetum glaucifolium*) [7]. Cultivation of improved forage varieties with better management practices and with supplementation of irrigation during the dry season is urgently needed, especially for dairy cattle [8]. Adie and Blümmel [9] pointed out that livestock fed improved forage crops grown through irrigation produce much more milk and meat, improving the diets of both their owners and their consumers.

Desho grass is among the improved forage crops in Ethiopia that could supply high‐quality forage for both smallholder farmers and intensive livestock production systems. It is highly palatable, nutritious, and fast‐growing, with a high leaf‐to‐stem ratio. The grass is also suitable for silage making, particularly for use during the dry season, and performs well at altitudes ranging from 1500 to 2800 masl [10, 11]. Due to its rapid growth and ability to be harvested frequently, desho grass is an effective option for alleviating feed shortages, especially during the dry season [12, 13]. It can produce biomass yields ranging from 30 to 110 t/ha [14]. Moreover, desho grass can yield 30–40 t/ha of dry matter even without fertilizer application and shows a positive response to fertilization [11]. In Ethiopia, a total of 75 improved forage varieties, along with their full production and utilization packages, have been released for different agroecologies [15].

Desho grass varieties provide many benefits for livestock; however, there is limited information on their adaptability and performance under dry‐season conditions. This knowledge gap hinders our understanding of how to effectively use these grasses to sustain animal nutrition and support agricultural practices in challenging conditions. A total of three varieties of desho grass (Areka/DZF #590, Kulumsa/DZF 592, and Kindu kosha‐1/DZF #591) have been registered for animal feed at the national level [16]. Then, studies on growth rates, biomass production, and the nutritional value of desho grass in response to supplementary irrigation in Ethiopia are lacking. This study is aimed at evaluating the agronomic performance, forage yield, and nutritive value of registered desho grass varieties under supplementary irrigation for livestock producers in western Ethiopia.

## 2. Materials and Methods

### 2.1. Description of the Study Area

The experiment was carried out under supplementary irrigation from October 24, 2022 to February 5, 2023, at the Dambi Dollo University in Efa Galano kebele, Sayo district of Kellem Wollega in Oromia Regional State, Western Ethiopia. The area lies at a latitude of 8°50 ^′^N and a longitude of 34°76 ^′^E and an altitude between 1500–1740 masl. It has a subhumid climate with average minimum and maximum annual temperatures of 15°C and 28°C, respectively. The area receives an annual rainfall of 850–1200 mm.

### 2.2. Weather Data

**Figure 1:**
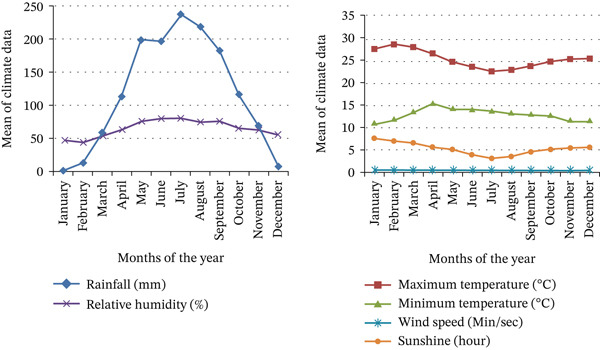
Ten years (2012–2021) weather data at Efa Galano kebele, Ethiopia. *Source:* Gambella Meteorological Agency, Gambella, Ethiopia.

Ten years (2012–2021) of average weather data (rainfall, minimum temperature, maximum temperature, relative humidity, wind speed, and sunshine) of the area were obtained from the Gambella meteorological agency. Accordingly, information on 10 years of weather data of the area is provided in Figure 1.

### 2.3. Soil Physicochemical Characteristics of the Study Site

Soil physicochemical characteristics of composite soil samples (0–30 cm depth) collected from the experimental site before planting are presented in Table 1. The collected soil sample was air‐dried, then subsampled, ground, and sieved through a 2‐mm mesh after removing plant debris. It was further milled to pass through a 0.2‐mm sieve for analyses of nitrogen, pH, organic carbon, available phosphorus, organic matter, cation exchange capacity (CEC), and soil texture [17]. Soil pH was measured with a digital pH meter in a 1:2.5 soil‐to‐distilled water suspension. Organic carbon was determined using the wet combustion method [18], and organic matter was calculated by multiplying % OC by 1.724. The CEC was analyzed with ammonium acetate, and available phosphorus was measured by shaking soil with 0.03 mole ammonium fluoride in 0.1 mole hydrochloric acid, per the Olsen II method [19]. The soil sample analysis was conducted at the Nekemte soil laboratory (Nekemte, Oromia, Ethiopia).

**Table 1:** Soil physicochemical characteristics.

Ser. No.	Parameters	Value
1	Ph	5.79
2	OC (g/kg)	26.1
3	OM (g/kg)	44.9
4	P (mg/kg)	7.82
5	TN (g/kg)	2.2
6	CEC (cmolc/kg)	32.06

Soil texture (g/kg)		
7	Clay	400
8	Silt	290
9	Sand	310

Texture class		
10	Clay loam	

Abbreviations: CEC, cation exchange capacity; OC, organic carbon; OM, organic matter; P, Olsen phosphorus; TN, total nitrogen.

### 2.4. Experimental Layout, Design, and Treatments

Three desho grass varieties (Areka/DZF #590, Kulumsa/DZF #592, and Kindu kosha‐1/DZF #591) were evaluated using randomized complete block design. To refine the soil, the land was plowed and harrowed with oxen, then hoed. The land was divided into four blocks, with a total of 12 plots. The plot size was (3 × 3 m) and the spacing between the plots and between blocks was 1 and 1.5 m, respectively. The SAS software (SAS, Version 9.3, Cary, North Carolina, United States) was used for the randomization of the treatments. The fine root bed plots were prepared before laying out the experimental plots. Vegetative root splits were used for planting desho grass, with row and plant spacing of 0.5 and 0.25 m, respectively, as per the recommendation [12] to form six rows per plot. Fertilizer was applied at a rate of 100 kg/ha NPS (N‐P‐K‐S, 19‐38‐0‐7) and 50 kg/ha (23% N) urea for all experimental units during establishment [20]. At various times throughout the experimental period, desho grass plots were manually weeded and forage grass growth was promoted through increased soil aeration [21].

### 2.5. Supplementary Irrigation System

The scheduling for irrigation using the CROPWAT model was carried out based on historical weather data obtained from the Gambella Meteorological Agency for a period of 10 years (Figure 1). In addition to the weather data, other important factors considered in the scheduling process were the soil characteristics of the study area, forage characteristics (such as crop coefficient and root depth), and dates of planting (October 24, 2022) and harvest for the forage crops (February 5, 2023) (Table 1). The crop coefficient (0.40 initial, 0.71 developmental, 0.89 middle, and 0.72 end phases) and root depth values (0.5 m) for desho grass were used as recommended in the literature [22]. The furrow irrigation method was used, and the irrigation schedule varied during different phases of the forage growth. During the initial and developmental phase, irrigation water was applied at intervals of 3 days. After the developmental phase, irrigation water was applied through furrows at about 5 days intervals until the end of the experimental phase. To measure the amount of water applied, a 7.5‐cm Parshall flume was used to measure discharges according to CROPWAT based on the crop water requirement schedule. During each irrigation water application, the set time and application time were monitored over time. The application time (min) required to irrigate the predetermined amount into each plot was calculated according to Kandiah [23]. During the experimental period, the gross and net irrigation amounts of water applied were 282.1 and 197.5 mm, respectively.

### 2.6. Data Collection

#### 2.6.1. Agronomic Parameters

Data on agronomic parameters were recorded (105 days after planting [DAP]). Prior to harvesting, the number of plants that survived in each plot was counted and converted into a survival percentage based on the initial plant population. Plant height, tillers/plant, nodules/plant, internode length, leaf length from the collar to tip, and leaf width were measured on five randomly selected plants from each plot. Leaves/tiller were counted on 10 plants. Data were averaged for each plot.

#### 2.6.2. Dry Matter Yield, Leaf‐to‐Stem Ratio, and Nutritive Value Determination

At 105 DAP, the harvest was carried out manually using a sickle at a stubble height of 8 cm [24]. First, the border rows were removed and two middle rows were harvested to measure the fresh weights. The fresh weight was immediately recorded in the field using a field balance. Fresh subsamples of forage were taken from each plot, weighed, and then chopped to a short length of 2–5 cm, oven dried at 65°C for 72 h, and reweighed to estimate DM content and yield. Then, the leaf‐to‐stem ratio was calculated by harvesting, mixing, and subsampling plants from each plot and separating the leaves and stems prior to oven drying (65°C for 72 h). The leaf‐to‐stem ratio was determined by dividing the leaf dry weight of the leaves by the stem dry weight.

The samples dried for DM and yield estimation were milled using a Wiley mill to pass through a 1‐mm sieve for chemical analysis. The AOAC method [25] was used to determine ash, and N. Crude protein (CP) was calculated as N × 6.25 [26]. Crude protein yield (CPY) was calculated as the product of DM yield with CP content divided by 100. The method of Van Soest et al. [27] was used to analyze neutral detergent fiber (NDF), acid detergent fiber (ADF), and acid detergent lignin (ADL). Hemicelluloses were calculated by subtracting ADF from NDF content, whereas cellulose was determined by subtracting ADL from ADF content. Determination of in vitro dry matter digestibility (IVDMD) was made using all of the samples that were used for chemical analysis using the Tilley and Terry [28] two‐stage method. Final stage samples were then ashed to estimate in vitro organic matter digestibility (IVOMD). The metabolizable energy (ME) content was estimated from IVOMD using the equation: ME (MJ kg − 1 DM) = 0.15 ∗ IVOMD [29]. All laboratory analyses were undertaken at Holeta Agricultural Research Center, Holeta, Oromia, Ethiopia.

### 2.7. Statistical Analysis

The collected data were subjected to the ANOVA procedure by using the general linear model of SAS software (SAS, Version 9.3, Cary, New Carolina, United States), requiring an alpha level of *p* < 0.05 for significant differences to test the effects of block, variety, and residual variance.

## 3. Results and Discussion

### 3.1. Agronomic Performance of Desho Grass Varieties

The results show significant variation (*p* < 0.05) among desho grass varieties in several agronomic traits, including survival rate, number of tillers per plant, leaves per tiller, total leaves, nodes per plant, internode length, leaf length, and leaf‐to‐stem ratio, except for plant height and leaf width (Table 2). There was a significant difference (*p* < 0.05) in the survival rate among desho grass varieties. The higher percentage of surviving plants was obtained from Areka/DZF #590 (99.77%), followed by Kulumsa/DZF #592 (98.38%), whereas the lowest survival rate was obtained from Kindu kosha‐1/DZF #591 (96.06%), with an overall mean of 98.06%. The survival rate in this study aligns with Wamatu [30], for desho grass. Based on current results, there was no significant difference (*p* > 0.05) in plant height between the varieties of desho grass. This aligns with earlier research by several authors [31, 32], which also found no notable variation in plant height among different desho grass varieties. In contrast, other studies have reported significant differences in plant height among these varieties [33]. Tillers density is crucial for grasses as it enhances endurance, forage quantity, and affects leaf area and DM yield. Significant differences (*p* < 0.05) in the number of tillers per plant were observed among varieties of desho grass.). The highest number of tillers per plant was found from Areka/DZF #590 (65.23), which was at par with Kulumsa/DZF #592 (63.57), whereas the lowest was recorded from Kindu kosha‐1/DZF #591 (55.63), with an overall mean of 61.48. Tiller production differences may arise from variety adaptability, as reported by Hidosa and Getaneh [31], whereas Wana et al. [34] noted no significant variation in similar desho grass varieties. This difference might be due to variations in soil fertility, harvesting stage, planting space, and climatic conditions.

**Table 2:** Agronomic performance of desho grass varieties.

Varieties	Parameters									
	SR (%)	PH (cm)	NTPP (count)	NLPT (count)	TNLPP (count)	NNPP (count)	IL (cm)	LL (cm)	LW (cm)	LSR
Areka/DZF #590	99.77^a^	84.28	65.23^a^	13.23^a^	854.39^a^	13.4^a^	9.97^a^	44.86^a^	1.68	0.79^a^
KK‐1/DZF #591	96.06^c^	78.96	55.63^b^	10.94^b^	606.34^b^	9.55^c^	8.76^b^	39.43^b^	1.59	0.69^c^
Kulumsa/DZF #592	98.38^b^	81.68	63.57^a^	12.89^a^	815.35^a^	12.43^b^	9.62^ab^	43.32^a^	1.62	0.76^b^
Overall mean	98.06	81.64	61.48	12.36	758.69	11.79	9.45	42.54	1.63	0.75
SE	0.40	2.46	2.03	0.38	23.39	0.25	0.31	1.31	0.04	0.02
*p* value	< 0.0001	0.3232	0.0048	0.0003	<0.0001	<0.0001	0.029	0.0182	0.46	0.0043
CV (%)	1.42	10.43	11.45	10.64	10.68	7.34	11.51	10.66	10.09	9.50

*Note:* Superscript letters (a–c) within a column mean they are statistically different (*p*<0.05).

Abbreviations: cm, centimeters; IL, internode length; KK‐1, Kindu kosha‐1/DZF #591; LL, leaf length; LSR, leaf‐to‐stem ratio; LW, leaf width; NLPT, number of leaves per tiller; NNPP, number of nodes per plant; NTPP, number of tillers per plant; PH, plant height; SE, standard error; SR, survival rate; TNLPP, total number of leaves per plant.

There was a significant difference (*p* < 0.05) in the number of leaves per tiller, total number of leaves per plant, and leaf length of desho grass among the tested varieties. The highest number of leaves per tiller was obtained from Areka/DZF #590 (13.23), which was insignificantly different from Kulumsa/DZF #592 (12.89), whereas the lowest count was observed from Kindu kosha‐1/DZF #591 (10.94). This finding surpasses earlier studies on the number of leaves per tiller of desho grass [24]. The differences in leaf counts are attributed to distinct phenotypic and genetic traits. The current result showed that Areka/DZF #590 (854.39) produced a higher total number of leaves per plant, which was at par with Kulumsa/DZF #592 (815.35), whereas Kindu kosha‐1/DZF #591 (606.34) was the lowest, with a mean total leaf count of 758.69. The total number of leaves per plant observed was greater than the figure reported by Asmare et al. [24] for desho grass at a similar harvesting age (105 DAP) under irrigation conditions. The greater number of leaves per plant enhances photosynthesis and promotes root growth, leading to increased organic matter accumulation [35]. Leaf length significantly influences the vegetative yield of forage grasses. The longest leaf was measured from Areka/DZF #590 (44.86 cm), which was at par with Kulumsa/DZF #592 (43.32 cm), whereas the shortest was measured from Kindu kosha‐1/DZF #591 (39.43 cm). The current mean leaf lengths align with Jabessa et al. [36] for similar desho grass but are longer than those reported by Tesfaye et al. [37] for 90 and 120 days postharvest. This among studies may be influenced by fertilizer rate, planting spacing, planting season, and management conditions.

The number of nodes per plant and internode length were significantly different (*p* < 0.05) among varieties of desho grass. The highest number of nodes per plant was recorded from Areka/DZF #590 *(*13.4*),* followed by Kulumsa/DZF #592 (12.43), and the lowest was counted from Kindu kosha‐1/DZF #591 (9.55), with an overall mean of 11.79. The observed values for the number of nodes per plant were higher than those reported by Kebede et al. [32] for similar desho grass varieties. This difference among studies and varieties might be as growth progresses and tillers increase; the apical meristem becomes indeterminate, enabling the specific variety to produce countless nodes and leaves, resulting in continuous growth of desho grass (Bantihun et al. [38]. The highest internode length was measured from Areka/DZF #590 (9.97 cm), followed by Kulumsa/DZF #592 (9.62 cm), whereas the lowest was measured from Kindu kosha‐1/DZF #591 (8.76 cm) with an overall mean of 9.45 cm. The current internode length finding contrasts with Kebede et al. [32], who reported no significant variation in internode length among desho grass varieties and noted lower mean values than those observed in this study. This could be attributed to several factors, including the season of the experiment, harvesting age, and altitude. The leaf width among varieties of desho grass did not vary significantly (*p* > 0.05). Numerically, the largest leaf width was measured from Areka/DZF #590 (1.68 cm), followed by Kulumsa/DZF #592 (1.62 cm) and Kindu kosha‐1/DZF #591 (1.59 cm), with an overall mean of 1.63 cm. Broad‐leaved varieties have a larger surface area that increases photosynthesis, resulting in more carbohydrates that promote leaf growth in width and length [39]. The measured leaf width per plant was higher than the findings reported by Bantihun et al. [38] for desho grass. The wider leaf is crucial for maximizing ground surface exposure and intercepting solar radiation during growth [40].

The leaf‐stem ratio was significantly different (*p* < 0.05) among varieties of desho grass. The highest leaf‐to‐stem ratio was obtained from Areka/DZF #590 (0.79), followed by Kulumsa/DZF #592 (0.76), whereas the lowest was from Kindu kosha‐1/DZF #591 (0.69) with an overall mean of 0.75. Similarly, the significant variation in the leaf‐to‐stem ratio among varieties of desho grass was reported by Kebede et al. [32], with a higher mean value than the present result. In contrast to the current result, no significant difference in leaf‐to‐stem ratio for similar desho grass varieties has been reported in various studies [33, 36]. This difference might be due to variations in soil, weather, and management conditions. The leaf‐to‐stem ratio is an indicator of nutritional quality, significantly influenced by the harvesting stage. A higher ratio indicates greater nutritive value, whereas a lower ratio suggests reduced value. The leaf‐to‐stem ratio in tropical forage grasses significantly influences ruminant diet selection, forage value, and intake [41].

### 3.2. Dry Matter Yield and Nutritive Value of Desho Grass Varieties

The agronomic characteristics, yield components, and nutritive value variables had a consistent pattern of differences across varieties with Areka > Kulumsa > KK − 1 because high CP and low fiber are more desirable [42] (Table 3). The results showed that all yield and nutritive value variables showed a significant difference among desho grass varieties, except hemicellulose. The DM content among varieties of desho grass did not vary significantly (*p* > 0.05). The lack of variation in dry matter content can be attributed to factors relating to the soil, the environment, and perhaps the physiological stage of the plant at the time of forage collection [43]. Differences among varieties for yield components (DMY, CPY) were consistent with those for agronomic characteristics, such that Areka > Kulumsa > Kindu kosha, which reflects its better nutrient utilizing response [44]. The highest dry matter yield was recorded from Areka/DZF #590 (12.64 t/ha), which was at par with Kulumsa/DZF #592 (11.63 t/ha), whereas Kindu kosha‐1/DZF #591 gave the lowest dry matter yield (9.17 t/ha). Desta [45] suggests that the Areka variety′s increased DMY is due to enhanced tiller number, leaf formation, and elongation, and stem development. The current DMY was lower than the values reported by previous scholars for similar desho grass varieties [31, 33, 36]. The CPY variability depends on DMY accumulation and CP content performance of the forage. In terms of CPY, the highest yield was obtained from Areka/DZF #590 (1.35 t/ha), followed by Kulumsa/DZF #592 (1.12 t/ha), whereas Kindu kosha‐1/DZF #591 (0.95 t/ha) gave the lowest CPY with an overall mean of 1.14 t/ha. The CPY in the current results exceeded the value found for desho grass [46]. The variation among studies may result from differences in soil fertility, weather, and management practices.

**Table 3:** Dry matter yield, crude protein yield, and nutritive value of desho grass varieties.

Varieties	DMY, CPY, and chemical composition							
	DM (g/kg)	DMY (t/ha)	CP (g/kg)	CPY (t/ha)	Ash (g/kg)	NDF (g/kg)	ADF (g/kg)	ADL (g/kg)
Areka/DZF #590	288.08	12.64^a^	107.3^a^	1.35^a^	93.2^a^	636^c^	393.9^c^	56.9^b^
KK‐1/DZF #591	279.84	9.17^b^	103.8^a^	0.95^c^	88.9^b^	659.8^a^	420.8^a^	62.3^a^
Kulumsa/DZF #592	290.80	11.63^a^	95.9^b^	1.12^b^	93.8^a^	648.4^b^	403.1^b^	64.0^a^
Overall mean	286.2	11.15	102.4	1.14	91.9	648.1	405.9	61.1
*p* value	0.25	< 0.0001	< 0.0001	< 0.0001	0.0346	< 0.0001	< 0.0001	0.0001
CV (%)	8.86	11.64	5.64	12.87	5.20	1.20	1.39	6.04
SE	5.76	0.37	1.67	0.04	1.38	2.25	1.62	1.06

*Note:* Superscript letters (a–c) within a column mean they are statistically different (*p*<0.05).

Abbreviations: ADF, acid detergent fiber; ADL, acid detergent lignin; CP, crude protein; CPY, crude protein yield; CV, coefficient of variation; DM, dry matter content; DMY, dry matter yield; KK‐1, Kindu kosha‐1/DZF #591; NDF, neutral detergent fiber; SE, standard error.

The higher CP content was recorded from Areka/DZF #590 (107.3 g/kg), which was at par with Kindu kosha‐1/DZF #591 (103.8 g/kg), whereas significantly lower CP content was obtained from Kulumsa/DZF #592 (95.9 g/kg) with an overall mean of 102.4 g/kg. The current CP content result was higher than in previous reports for desho grass harvested at different ages [24]. The CP content of forage crops is greatly influenced by variety [38]. Desho grass varieties tend to have higher CP content than most tropical forage grasses, likely due to a greater leaf‐to‐stem ratio at harvest. The current CP content obtained from desho grass varieties is above the minimum CP requirements (70 g/kg) for the maintenance of animals and rumen microbes [41] and with the range of most CP content for tropical pasture, being between 70 and 120 g/kg [47]: however, it is lower than the recommended CP between 140 and 160 g/kg for sustainable production of dairy cattle [48].

Ash refers to the total mineral content of the forage. The higher ash content of *desho* grass was obtained from Kulumsa/DZF #592 (93.8 g/kg), whereas the lowest was obtained from Kindu kosha‐1/DZF #591 (88.9 g/kg). The overall mean ash content was 91.9 g/kg. The current ash contents were lower than the previous report Jabessa et al. [36] for similar desho grass varieties tested at the midland and highland Guji zone. The genetic ability to absorb minerals and its mineral requirements for growth significantly affect ash concentration [44]. Hence, producers should pay attention to minerals because they lower forage intake, reduce digestibility, negatively affect fermentation, and dilute forage nutritive value [44]. Poor‐quality forage will simply take up space in a cow’s stomach, not delivering nutritional value and declining milk production [44]. Generally, mineral concentration decreases as plants mature and is greater in forages grown in soils that contain high concentrations of available minerals [38].

The NDF content of feed has a detrimental effect on forage intake [49]. The studied varieties might have various genetic characteristics that contributed to the variability in NDF content. The significantly higher (*p* < 0.05) NDF content in the current result was obtained from Kindu kosha‐1/DZF #591 (659.8 g/kg) followed by Kulumsa/DZF #592 (648.4 g/kg), whereas lower NDF content was recorded from Areka/DZF #590 (636 g/kg) with an overall mean of 648.1 g/kg. The overall mean of the current results was comparable to Wamatu [30] findings on the NDF content of desho grass. The feed that contains 45% to 65% NDF is considered a moderate level of quality, whereas feed containing more than 65% NDF is classified as roughages of low quality [50]. Based on this classification, Kindu kosha‐1/DZF #591 is considered poor‐quality feed, whereas Kulumsa/DZF #592 and Areka/DZF #590 can be classified as medium quality. The current values are above the recommended NDF (250–330 g/kg) needed for lactating cows [48].

The significantly higher (*p* < 0.05) ADF content was obtained from Kindu kosha‐1/DZF #591 (420.8 g/kg) followed by Kulumsa/DZF #592 (403.1 g/kg), whereas significantly lower ADF was recorded from Areka/DZF #590 (393.9 g/kg). The current ADF values were lower than the previous report for similar desho grass varieties [36]. The current result was under the range between 300 and 450 g/kg of the majority ADF content for tropical pasture species [51]. Kellems and Church [52] stated that roughages with an ADF content higher than 400 g/kg are considered low quality, whereas those with less than 400 g/kg are considered high quality. Based on this classification, Kindu kosha‐1/DZF #591 and Kulumsa/DZF #592 would be classified as poor quality, whereas Areka/DZF #590 would be classified as medium quality. However, the current result was above the recommended ADF (170–210 g/kg) recommended for lactating cows [48]. The significantly higher (*p* < 0.05) ADL content was obtained from Kulumsa/DZF #592 (64 g/kg), which was at par with Kindu kosha‐1/DZF #591 (62.3 g/kg), whereas significantly lower ADF was recorded from Areka/DZF #590 (56.9 g/kg). The ADL values in this study were lower than the reported values, which ranged from 6.47% to 12.03% and 8.8% to 26.7% in Guji midland and highland areas for similar *desho* grass varieties, respectively [36]. However, it was slightly higher than the values reported by Kebede et al. [32] which ranged from 4.3% to 4.5% for similar *desho* grass varieties. Van Soest [53] found that lignin content above 60 g/kg has a negative impact on the digestibility of forage. Based on this, only the Areka/DZF #590 variety had lignin content below this threshold, resulting in less impact on the digestibility of ruminants.

### 3.3. In Vitro Digestibility and ME Content of Desho Grass Varieties

The highest dry matter digestibility was obtained from Areka/DZF #590 (624.7 g/kg), followed by Kulumsa/DZF #592 (584.3 g/kg), whereas the minimum was obtained from Kindu kosha‐1/DZF #591 (580.9 g/kg) with an overall mean of 596.6 g/kg. The current results (Table 4) for IVDMD of Kindu kosha‐1/DZF #591 and Kulumsa/DZF #592 were lower than those previously reported, whereas Areka/DZF #590 showed higher digestibility [32]. The present result lies within the 400–700 g/kg IVDMD range for grasses found in tropical and subtropical areas [54]. The current %IVDMD values are above 450 g/kg, which is the required level for maintenance cattle in the tropics [55]. However, the present result is lower than the threshold reported by Rivera and Parish [56], who noted that IVDMD greater than 650 g/kg indicates good feeding value, and values below this threshold level result in reduced intake. Therefore, the current result implicates lower voluntary intake.

**Table 4:** In vitro digestibility and metabolizable energy of desho grass varieties.

Varieties	Parameters		
	IVDMD (g/kg)	IVOMD (g/kg)	ME (MJ/kg^−1^)
Areka/DZF #590	624.7^a^	608.4^a^	9.13^a^
Kindu kosha‐1/DZF #591	580.9^b^	576.1^b^	8.64^b^
Kulumsa/DZF #592	584.3^b^	559.1^c^	8.39^c^
Overall mean	596.6	581.2	8.72
*p* value	< 0.0001	< 0.0001	< 0.0001
CV (%)	1.63	3.49	3.49
SE	0.28	0.59	0.09

*Note:* Superscript letters (a–c) within a column mean they are statistically different (*p*<0.05).

Abbreviations: CV, coefficient of variation; IVDMD, in vitro dry matter digestibility; IVOMD, in vitro organic matter digestibility; kg, kilogram; ME, metabolizable energy; MJ, megajoule; SE, standard error.

A IVOMD significant difference (*p* < 0.05) was observed among varieties of desho grass. The highest was obtained from Areka/DZF #590 (608.4 g/kg), followed by Kindu kosha‐1/DZF #591 (576.1 g/kg), whereas the minimum was obtained from Kulumsa/DZF #592 (559.1 g/kg) with the overall mean of 581.2 g/kg. This difference could be due to the performance of a variety under the same managemental condition. These findings are consistent with Wamatu [30] for the IVOMD of desho grass but lower than the IVOMD for different tropical grass reported by Chapman [57]. This difference among studies could be due to forage species. The present %IVOMD values were observed above the critical threshold level of 500 g/kg required for feeds to be considered as having acceptable digestibility [58]. Regarding ME, a significant difference (*p* < 0.05) was observed between desho grass varieties. The maximum ME was recorded from Areka/DZF #590 (9.13 MJ/kg^−1^) followed by Kindu kosha‐1/DZF #591 (8.64 MJ/kg^−1^), whereas the minimum was recorded from Kulumsa/DZF #592 (8.39 MJ/kg^−1^) with an overall mean 8.72 MJ/kg^−1^. The current result was relatively comparable with the report of Mengistu [59], who noted that the ME content of desho grass was 8.22 MJ/kg, and with the result of Wamatu [30], who stated that the ME content of sole desho grass was 8.12 MJ/kg. The current finding reveals that Kindu kosha‐1/DZF #591 and Kulumsa/DZF #592 are classified as low energy feeds (<9 MJ/kg), whereas Areka/DZF #590 is considered as medium energy (> 9 MJ/kg) feed based on the classification reported by Lonsdale [60] for the ME content of feedstuffs. The current ME values obtained were lower than the acceptable range for cattle, sheep, and some classes of dairy cattle (9.97–10.52 MJ/kg) [61].

## 4. Conclusions

All desho grass varieties performed well at the experimental site, but they varied in terms of several agronomic characteristics, chemical composition, and in vitro digestibility. Based on their high herbage dry matter production potential and nutritive value at 105 DAP, Areka/DZF/DZF #590 were chosen as improved forage varieties suitable for animal feeds in the livestock industry under supplementary irrigation conditions. Further research is needed on animal performance with these varieties to provide firm recommendations.

## Author Contributions

**Fikre Dereba:** writing – review & editing, writing – original draft, software, supervision, methodology, investigation, formal analysis, data curation, conceptualization. **Zemene Worku:** writing – review & editing, supervision, investigation, data curation, conceptualization. **Diriba Geleti:** writing – review & editing, supervision, data curation, conceptualization.

## Funding

No funding was received for this manuscript.

## Ethics Statement

The authors have nothing to report.

## Conflicts of Interest

The authors declare no conflicts of interest.

## Data Availability

The data that support the findings of this study are available from the corresponding author upon reasonable request.

## References

[1] CSA , Agricultural Sample Survey 2020/21 Report on Livestock and Livestock Characteristics, Volume II Statistical Bulletin 589, 2021, Central Statistical Authority.

[2] Duguma B. and Janssens G. P. , Assessment of Livestock Feed Resources and Coping Strategies With Dry Season Feed Scarcity in Mixed Crop-Livestock Farming Systems Around the Gilgel Gibe Catchment, Southwest Ethiopia, Sustainability. (2021) 13, no. 19, 10713, 10.3390/su131910713.

[3] Mekuriaw Z. and Harris-Coble L. , Ethiopia’s Livestock Systems: Overview and Areas of Inquiry, 2021, Feed the Future Innovation Lab for Livestock Systems, https://hdl.handle.net/10568/116578.

[4] Boote K. J. , Adesogan A. T. , Balehegn M. , Duncan A. , Muir J. P. , Dubeux J. C. , and Rios E. F. , Fodder Development in Sub-Saharan Africa: An Introduction, Agronomy Journal. (2022) 114, no. 1, 1–7, 10.1002/agj2.20924.

[5] Mengistu A. , Kebede G. , Feyissa F. , and Assefa G. , Review on Major Feed Resources in Ethiopia: Conditions, Challenges and Opportunities, Academic Research Journal of Agricultural Science and Research. (2017) 5, no. 3, 176–185, 10.14662/ARJASR2017.013.

[6] Feyissa F. , Kebede G. , Geleti D. , Assefa G. , and Mengistu A. , Improved Forage Crops Research and Development in Ethiopia: Major Achievements, Challenges and the Way Forward, OMO International Journal of Sciences. (2022) 5, no. 2, 36–69, 10.59122/135BE51.

[7] Worqlul A. W. , Dile Y. T. , Bezabih M. , Adie A. , Srinivasan R. , Lefore N. , and Clarke N. , Identification of Suitable Areas for Fodder Production in Ethiopia, Catena. (2022) 213, 106154, 10.1016/j.catena.2022.106154.

[8] Mijena Jibat D. , Effect of NPS Fertilizer and Harvesting Stage on Agronomic Performance and Nutritional Quality of Bracharia Grass Under Supplementary Irrigation at Wondogenet, Southern Ethiopia, 2022, Doctoral dissertation, Ambo University.

[9] Adie A. and Blümmel M. , Irrigated Forages Improve Livestock Productivity and Livelihoods in Ethiopia, 2019, https://hdl.handle.net/10568/100486.

[10] Leta G. , Duncan A. J. , and Abdena A. , Desho Grass (Pennisetum pedicellatum) for Livestock Feed, Grazing Land and Soil and Water Management on Small-Scale Farms, 2013, NBDC Brief, https://hdl.handle.net/10568/33316.

[11] Wamatu J. , Desho Grass (*Pennisetum pedicellatum*), a Feed Resource for Mid and High Altitude Regions of Ethiopia, 2016, https://hdl.handle.net/20.500.11766/6167.

[12] Bedeke W. , Hidosa D. , and Berhanu T. , Effect of Planting Space and Fertilizer Rate on Productivity of Desho Grass (*Pennisetum pedicellatum*) in Jinka Agricultural Research Center, Southern Ethiopia, International Journal of Research in Agriculture and Forestry. (2017) 4, no. 11, 14–19.

[13] Beyene M. A. , Production Status, Biomass Yield Under Different Management Practices and Nutritional Values of Desho Grass (Pennisetum pedicellatum) in Southern Ethiopia, 2021, Doctoral Dissertation, Hawassa University.

[14] Asmare B. , Demeke S. , Tolemariam T. , Tegegne F. , and Wamatu J. , The Potential of Desho Grass (*Pennisetum pedicellatum Trin*.) for Animal Feed and Land Management Practices in Ethiopia: A Review, Global Journal of Animal Scientific Research. (2018) 6, no. 1, https://hdl.handle.net/10568/98481.

[15] MoA , Plant Variety Release, Protection and Seed Quality Control Directorate, 2021, Ministry of Agriculture, Crop Variety Register Issue No. 24.

[16] MOA , Plant Variety Releasing, Protection and Seed Quality Control Directorate, 2017, Ministry of Agriculture, Crop Variety Register. Issue No. 20.

[17] Van Reeuwijk L. P. , Procedures for Soil Analysis.Technical Paper, 2002, 9, 6th edition, International Soil Reference and Information Centre.

[18] Walkley A. and Black I. A. , An Examination of the Degtjareff Method for Determining Soil Organic Matter, and a Proposed Modification of the Chromic Acid Titration Method, Soil Science. (1934) 37, no. 1, 29–38, 10.1097/00010694-193401000-00003.

[19] Olsen S. R. , Estimation of Available Phosphorus in Soils by Extraction With Sodium Bicarbonate (No. 939), 1954, US Department of Agriculture.

[20] Danano D. , Liniger H. and Critchley W. , Improved Grazing Land Management- Ethiopia, Where the Land Is Greener, 2007, WOCAT, 313–316.

[21] Orodho A. , The Role and Importance of Napier Grass in the Smallholder Dairy Industry in Kenya, 2006, Food and Agriculture Organization.

[22] Mengistu D. T. , Evaluation of Wetting Front Detector to Determine Water Demand, Water and Crop Productivity of Selected Fodder Varieties Under Supplimental Irrigation (Case Studies in Lemo and Angeacha Areas of SNNP Region), 2015, Master′s thesis, Arba Minch University). National Academic Digital Repository of Ethiopia.

[23] Kandiah A. , Guide for Measurement of Irrigation Water Using Parshall Flumes and Siphons. FAO Irrigation and Drainage Paper No. 26, 1981, Food and Agriculture Organization of the United Nations.

[24] Asmare B. , Mekuriaw Y. , and Tekliye L. , Evaluation of Desho Grass (*Pennisetum pedicellatum Trin.*) Morphology, Yield and Chemical Composition Under Irrigation in Northwestern Ethiopia, Journal of Agriculture and Environment for International Development. (2018) 112, no. 2, 241–252, 10.12895/jaeid.20182.704.

[25] AOAC , Official Methods of Analysis, 1990, 15th edition, Association of Analytical Chemists Inc.

[26] Magomya A. M. , Kubmarawa D. , Ndahi J. A. , and Yebpella G. G. , Determination of Plant Proteins via the Kjeldahl Method and Amino Acid Analysis: A Comparative Study, International Journal of Scientific & Technology Research. (2014) 3, no. 4, 68–72.

[27] Van Soest P. J. , Roberston J. B. , and Lewis B. A. , Methods for Dietary Fiber, Neutral Detergent Fiber, and Non Starch Polysaccharides in Relation to Animal Nutrition, Journal of Dairy Science. (1991) 74, 3583–3597, 10.3168/jds.S0022-0302(91)78551-2. 1660498

[28] Tilley J. M. A. and Terry R. A. , A TWO‐STAGE Technique for THEIN VITRODIGESTION of Forage Crops, Journal of British Grassland Society. (1963) 18, no. 2, 104–111, 10.1111/j.1365-2494.1963.tb00335.x.

[29] Pinkerton B. , Forage Quality. Clemson University Cooperative Extension Service. Forage Fact Sheet 2, 2005, Cooperative Extension Service, Clemson University.

[30] Wamatu J. , Farmers′ Participatory Evaluation and Performance Testing of Selected Forage Varieties in Selected Districts of Eastern Amhara Region, Ethiopia, 2021, International Center for Agricultural Research in the Dry Areas (ICARDA).

[31] Hidosa D. and Getaneh D. , Evaluation of Desho (*Pennisetum pedicellatum*) Grass Varieties for Dry Matter Yield and Chemical Composition Under Irrigation in Two Districts of South Omo Zone, Southwestern Ethiopia, East African Journal of Sciences. (2021) 15, no. 1, 71–78.

[32] Kebede G. , Faji M. , Feyissa F. , Mohammed K. , Mengistu G. , Dejene M. , Geleti D. , Assefa G. , Mengistu S. , and Tsegahun A. , Biomass Yield and Chemical Composition of Desho Grass (*Pennisetum glaucifolium*) Varieties in Central Highlands of Ethiopia, Livestock Research Results. (2022) 10, 10.13140/RG.2.2.28666.81603.

[33] Gadisa B. , Dinkale T. , and Debela M. , Evaluation of Desho Grass (*Pennisetum pedicellatum* Trin) Lines for Their Adaptability at Mechara Research Station, Eastern Oromia, Ethiopia, Journal of Ecology and The Natural Environment. (2019) 11, no. 3, 26–32, 10.5897/JENE2019.0742.

[34] Wana D. , Husen N. , and Abate D. , Evaluation of Desho (*Pennisetum pedicellatum Trin*) Grasses for Dry Matter Yield and Nutritive Quality for the Mid Rift Valley of Oromia at Adami Tulu Agricultural Research Center, Journal of Scientific and Innovative Research. (2021) 10, no. 2, 49–52, 10.31254/jsir.2021.10204.

[35] Xu Z. and Zhou G. , Responses of Leaf Stomatal Density to Water Status and Its Relationship With Photosynthesis in a Grass, Journal of Experimental Botany. (2008) 59, no. 12, 3317–3325, 10.1093/jxb/ern185. 18648104 PMC2529243

[36] Jabessa T. , Bekele K. , and Amare Z. , Evaluation of Desho Grass for Their Agronomic Performances and Nutritive Values in Highland and Midland Areas of Guji Zone, Southern Oromia, Ethiopia, Science Research. (2021) 9, no. 3, 10.11648/j.sr.20210903.11.

[37] Tesfaye T. , Asmare B. , Mekuriaw Y. , and Hunegnaw B. , Morphological Characters, Yield, and Chemical Composition Potentials of Desho Grass (*Pennisetum glaucifolium H*.) Intercropped with Vetch Species in the Highlands of Ethiopia, Advances in Agriculture. (2022) 2022, 10.1155/2022/7874717, 7874717.

[38] Bantihun A. , Asmare B. , and Mekuriaw Y. , Comparative Evaluation of Selected Grass Species for Agronomic Performance, Forage Yield, and Chemical Composition in the Highlands of Ethiopia, Advances in Agriculture. (2022) 2022, 10.1155/2022/6974681, 6974681.

[39] Wilson J. R. , Hacker J. B. , Environmental and Nutritional Factors Affecting Herbage Quality, Nutritional Limits to Animal Production From Pastures, 1982, Commonwealth Agricultural Bureaux, 111–131.

[40] Zhang Z. , Christensen M. , Nan Z. , Whish J. P. , Bell L. W. , Wang J. , Wang Z. , Sim R. , and Sim R. , Plant Development and Solar Radiation Interception of Four Annual Forage Plants in Response to Sowing Date in a Semi-Arid Environment, Industrial Crops and Products. (2019) 131, 41–53, 10.1016/j.indcrop.2019.01.028.

[41] Minson D. J. , Forage in Ruminant Nutrition, 1990, Academic Press.

[42] Eshetu N. , Effect of Harvesting Dates on Biomass Yield and Nutritive Value of Improved Desho Grass (Pennisetum glucifolium) Varieties in Central Highlands of Ethiopia, 2024, Debre Berhan University (DBU), https://hdl.handle.net/10568/159687.

[43] Wekgari Y. , Tolemariam T. , and Worku Z. , The Effects of Intercropping Two Vetch Species (*Vicia sativa* and *Vicia dasycarpa*) at Different Row Spacing on Yield and Nutritional Values of Desho Grass (*Pennisetum glaucifolium*) in Sayo District, Western Ethiopia, 2020, https://repository.ju.edu.et//handle/123456789/5705.

[44] Kebede G. , Feyissa F. , Faji M. , Mohammed K. , Dejene M. , Mengistu G. , Geleti D. , Assefa G. , Alemayehu M. , Mengistu S. , and Mengistu A. , Dry Matter Accumulation Dynamics, Morphological Characteristics and Nutritive Value of Desho (*Pennisetum glaucifolium*) Grass Varieties in the Central Highlands of Ethiopia, Journal of Agriculture and Environmental Sciences. (2023) 8, no. 1, 110–123, 10.20372/jaes.v8i1.1479.

[45] Desta A. G. , Dry Matter Yield of Desho Grass (*Pennisetum pedicellatum*) Varieties, SustainableEnvironment. (2024) 10, no. 1, 2345435, 10.1080/27658511.2024.2345435.

[46] Faji M. , Kebede G. , Feyissa F. , Mohammed K. , and Mengistu G. , Yield, Yield Components, and Nutritive Value of Perennial Forage Grass Grown Under Supplementary Irrigation, Advances in Agriculture. (2022) 2022, 5471533, 10.1155/2022/5471533.

[47] Aminah A. and Chen C. P. , Future Prospects for Fodder and Pasture Production, Feeding Dairy Cows in the Tropics, Rome: Food and Agriculture Organization, 1998, Daya Publishing, 127–141.

[48] National Research Council , Nutrient Requirements of Dairy Cattle, 2001, 519, National Research Council.

[49] Van Soest P. J. , Nutritional Ecology of Ruminants, 1994, 2nd edition, Cornell University Press.

[50] Rajupreti C. , Nutrient Content of Feeds and Fodder in Nepal, 2006, 1st edition, Edition printed by Nirav Printing and General Order Suppliers.

[51] Jayasinghe P. , Ramilan T. , Donaghy D. J. , Pembleton K. G. , and Barber D. G. , Comparison of Nutritive Values of Tropical Pasture Species Grown in Different Environments, and Implications for Livestock Methane Production: A Meta-Analysis, Animals. (2022) 12, no. 14, 10.3390/ani12141806, 35883354. PMC9311783 35883354

[52] Kellems R. O. and Church D. C. , Livestock Feeds & Feeding, 1998, 4th edition, PrenticeHall, Inc.

[53] Van Soest P. J. , Analytical Systems for Evaluation of Feeds, Nutritional Ecology of the Ruminant, 1982, O&B Books, 75–94.

[54] Van Soest P. J. and Robertson J. B. , Analysis of Forages and Fibrous Foods, 1985, Cornell University.

[55] Youngquist J. B. , Carter D. C. , and Clegg M. D. , Grain and Forage Yield and Stover Quality of Sorghum and Millet in Low Rainfall Environments, Experimental Agriculture. (1990) 26, no. 3, 279–286, 10.1017/S0014479700018433.

[56] Rivera J. D. and Parish J. A. , Interpreting Forage and Feed Analysis Reports, 2010, Mississippi State University Extension Service.

[57] Chapman P. G. , Protein Degradation in the Rumen of Sheep Fed Pangola Grass and Siratro Hay, 1986, Master′s Thesis School of Land, Crop and Food Sciences, the University of Queensland, 10.14264/5b2dd18.

[58] Owen E. and Jayasuriya M. C. N. , Use of Crop Residues as Animal Feeds in Developing Countries, Research and Development in Agriculture. (1989) 6, no. 3, 129–138.

[59] Mengistu S. H. I. M. E. L. I. S. , Assessment of Forage Production, Feed Resource Utilization and Substitution Effect of Oat-Vetch Forage for Concentrate Mix on Performance of Sheep Fed Desho Grass as a Basal Diet in Damote Gale District of Wolaita Zone, SNNPR, 2018, Doctoral dissertation, Hawassa University.

[60] Lonsdale C. , Straights: Raw Materials for Animal Feed Compounders and Farmers, 1989, Scholium International.

[61] Marie-Madeleine N. P. , Kouassi D. , Magloire K. I. , Doulaye K. , Amougou A. , Jean B. , and Bassirou B. , In Vitro Gas Production and Digestibility of *Echinochloa Pyramidalis (Chase) Hitchc*. and Chase Grown under Constructed Wetland Treating Faecal Sludge as Ruminant Feed, International Journal of Scientific & Engineering Research. (2012) 3, no. 12, 1–6.

